# Effects of catheter sizing on pressure gradient measurements in a model of vessel stenosis

**DOI:** 10.1186/s42155-026-00681-z

**Published:** 2026-05-04

**Authors:** Duygu Harmankaya, Kay Pieterman, Eline van der Hoek, Maryam Hussain, Fleur van den Bogaert, Kirsten Henken, Marco Bruno, Desirée van Noord

**Affiliations:** 1 Department of Gastroenterology & Hepatology, Erasmus MC University Medical Centre, Rotterdam, the Netherlands; 2 Department of Radiology & Nuclear Medicine, Erasmus MC University Medical Centre, Rotterdam, the Netherlands; 3 Department of Gastroenterology & Hepatology, Franciscus, Rotterdam, the Netherlands; 4 Delft University of Technology, Delft, the Netherlands; 5 Department of Clinical Physics, Franciscus, Rotterdam, the Netherlands

**To the editor**

Chronic mesenteric ischemia (CMI) is a condition often underdiagnosed, typically presenting as postprandial abdominal pain resulting in fear of eating and weight loss [1–4]. The primary cause of CMI is mesenteric artery stenosis (MAS), commonly resulting from atherosclerosis in the celiac artery (CA), superior mesenteric artery (SMA), or inferior mesenteric artery. Despite severe atherosclerosis, some individuals remain asymptomatic as collateral pathways gradually develop in response to the ongoing atherosclerosis.

For patients with significant multi-vessel stenosis, percutaneous mesenteric artery stenting (PMAS) is the treatment of choice. The benefit of PMAS in patients with borderline stenosis or well-developed collateral circulation pathways remains uncertain, and overtreatment occurs in 27–31% of cases. Careful selection of patients is therefore essential. An objective tool to assess the hemodynamic significance of a stenosis while accounting for collateral circulation is highly valuable.

Fractional flow reserve (FFR), which measures the pressure gradient across a narrowing during angiography is widely used in coronary interventions, but its application in mesenteric artery procedures lacks consensus. Pressure measurements during vascular interventions are typically performed with 4/5 French angiography catheters. A previous study showed that pressure gradient measurements after administering a vasodilator were able to predict symptom relief following PMAS, with an 86% sensitivity and 83% specificity [5–11].

The goal of this study is to compare the performance of macrocatheters, microcatheters, and pressure wires in measuring pressure gradients in various degrees of stenosis in the CA and SMA, using an in-vitro model.

A 4-French macrocatheter, 2.4-French microcatheter, and 0.014-inch pressure wire were used. A Dräger Infinity C500 system was used for catheter pressure measurements and Philips Volcano Core Mobile device for pressure wire measurements (Table 1). An in-vitro model simulated the CA and SMA using silicone tubes, with flow rates of 1400 ml/min for the SMA and 1100 ml/min for the CA to simulate the postprandial state. Fluid pressure was set to 80 mmHg for the CA and 30 mmHg for the SMA (Fig. 1).

**Table 1:** Characteristics of the three different instruments used in this study

	Macro-catheter	Micro-catheter	Pressure wire
Brand and type	Cordis MPA	Terumo Progreat	Philips Omniwire
Diameter	4 French	2.4 French	0.36 mm
Length	125 cm	125 cm	185 cm
Solid/hollow	Hollow	Hollow	Solid
Read-out device	Dräger Infinity C500	Dräger Infinity C500	Philips Volcano Core Mobile
Position of sensor	Pressure transducer at the end of the wire	Pressure transducer at the end of the wire	Pressure sensor 3 cm proximal to the tip of the wire
Costs	€15,00	€216,00	€280,00

**Fig. 1:**
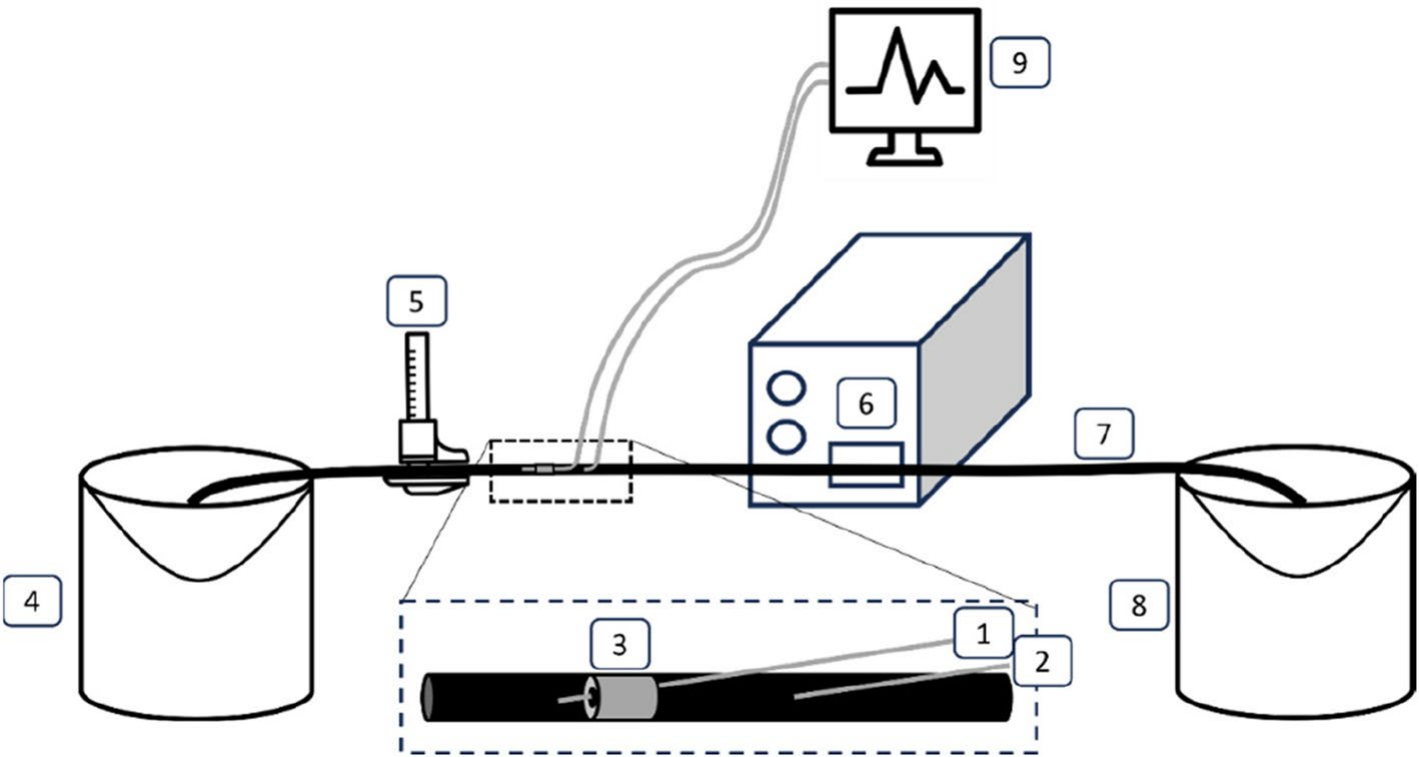
Schematic representation of the in-vitro model simulating the CA and SMA in post-prandial state. 1: distal instrument, 2: proximal instrument, 3: stenosis, 4: water collection reservoir, 5: calliper, 6: peristaltic pump system, 7: silicone tube, 8: water reservoir

Twenty-four 3-d printed stenoses were created of varying lengths and degrees (Table 2, Fig. 2). FFR was used to assess the hemodynamic significance of each stenosis. An FFR value of 0.80 or below is typically considered clinically significant for stenosis. FFR was calculated by measuring the pressure with various pump flow rates and stenosis types with each of the three instruments.

**Table 2:** Overview of the different stenoses with their inner radius, area and corresponding degree of stenosis

Inner diameter (mm)	Area (mm^2^)	Stenosis (%)
2	3.14	92
3	7.06	82
3.5	9.62	75
4	12.56	67
4.5	15.90	59
5	19.63	49

**Fig. 2:**
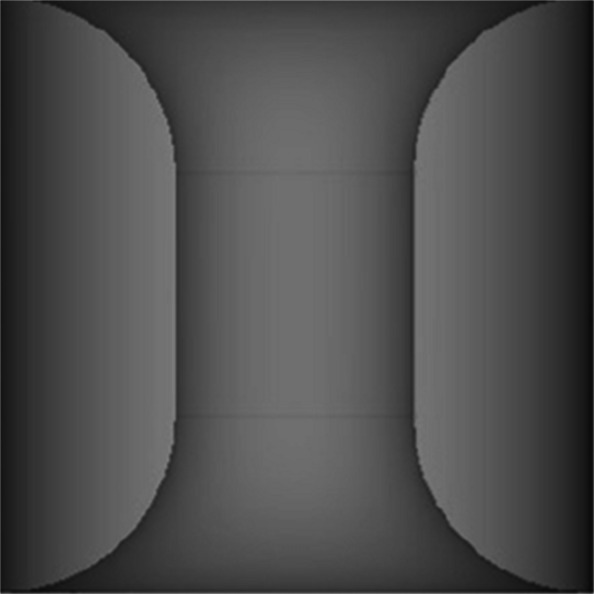
Design of one of the stenosis

All devices exhibited a clear inverse relationship between stenosis severity and FFR values (Tables 3 and 4). However, the pressure wire showed more consistent readings across all stenosis degrees, with less variability. Macrocatheters overestimated FFR for severe stenoses (> 75%) due to (near) wedge position of the catheter (Figs. 3 and 4). In clinical care, cautious interpretation of measurements using macrocatheters in high-grade stenoses is therefore recommended.

**Table 3:** Pressure measurements of microcatheter, macro-catheter and pressure wire during different flow rates

Flow (ml/min)	Pressure (mmHg)		
	Macro-catheter	Micro-catheter	Pressure wire
900	0	0	0
1000	6	6	2
1100	11	11	9
1200	25	25	16
1300	43	44	35
1400	66	66	61

**Table 4:** Pressure measurements of microcatheter, macro-catheter and pressure wire during different flow rates

Devices compared	Vessel	Pearson’s Correlation	*P*-value
Macro vs Micro	CA	.753	**< 0.001**
Macro vs Micro	SMA	.547	**< 0.001**
Macro vs Pressure wire	CA	.020	.89
Macro vs pressure wire	SMA	.079	.60
Micro vs Pressure wire	CA	.045	.79
Micro vs Pressure wire	SMA	.254	.09

**Fig. 3:**
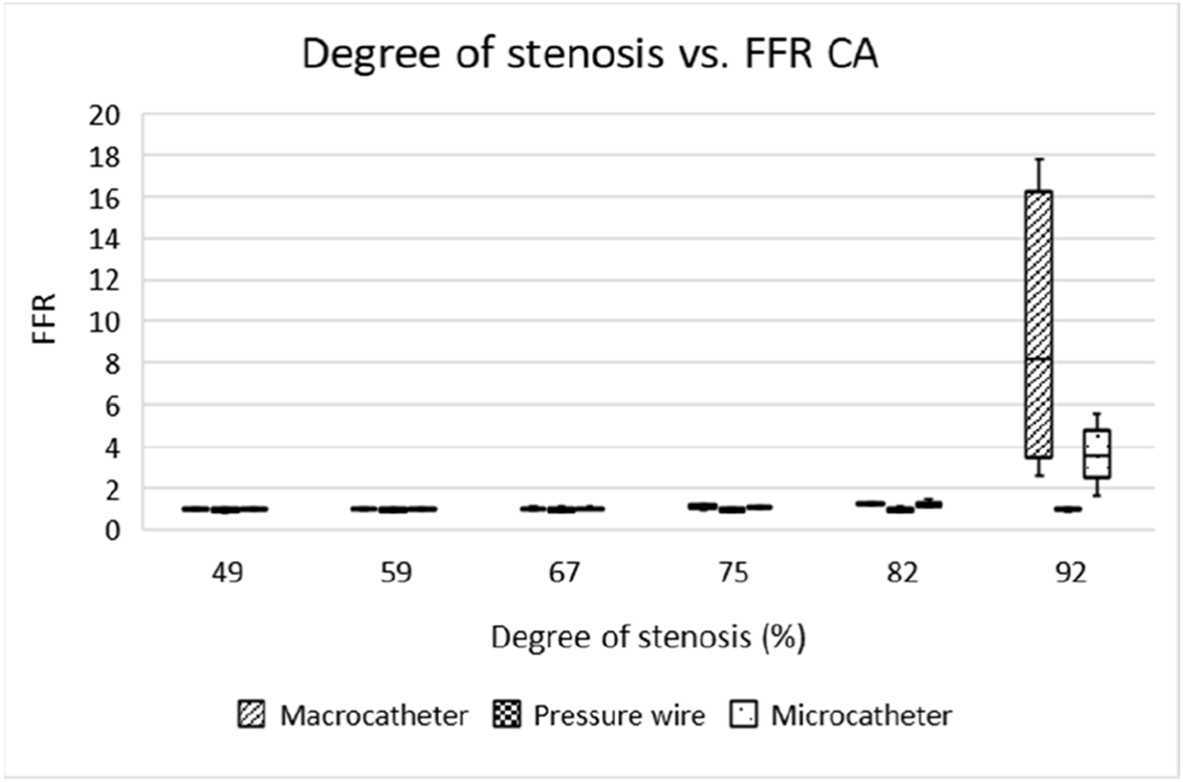
Box-and-whisker plot showing the distribution and variability of FFR measurements across different degrees of stenosis in the coeliac artery. FFR = fractional flow reserve, CA = coeliac artery

**Fig. 4:**
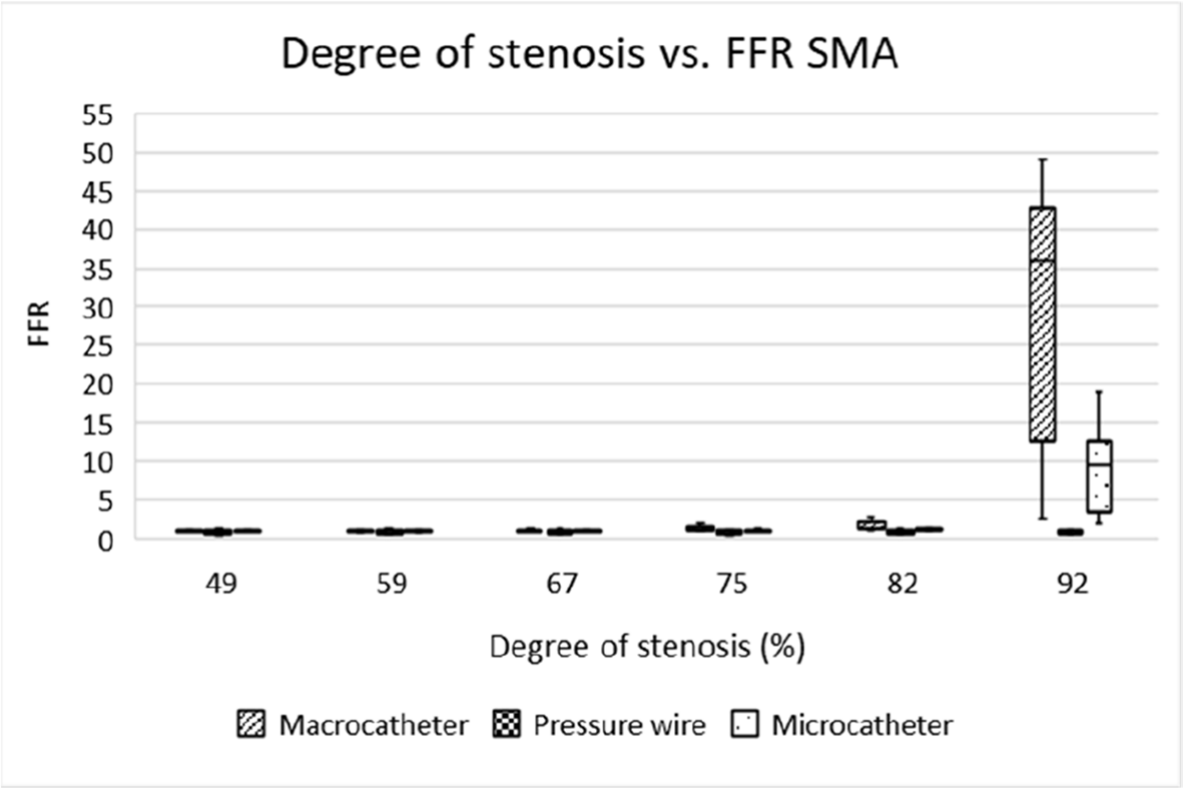
Box-and-whisker plot showing the distribution and variability of FFR measurements across different degrees of stenosis in the superior mesenteric artery. FFR = fractional flow reserve, SMA = superior mesenteric artery

Microcatheters and pressure wires showed comparable utility for assessing stenosis of all degrees, with microcatheters being more practical in clinical settings due to their lower cost and good availability in most centers. Pressure wires, while offering more precise measurements, are expensive and require specialized equipment (Figs. 5, 6, 7, 8, 9 and 10).

**Fig. 5:**
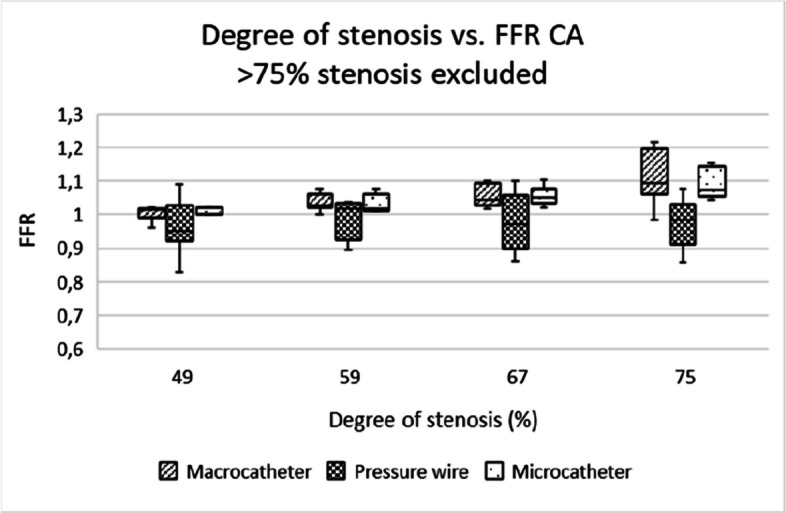
Box-and-whisker plot showing the distribution and variability of FFR measurements across different degrees of stenosis in the coeliac artery, when excluding the 82% and 92% stenosis. FFR = fractional flow reserve, CA = coeliac artery

**Fig. 6:**
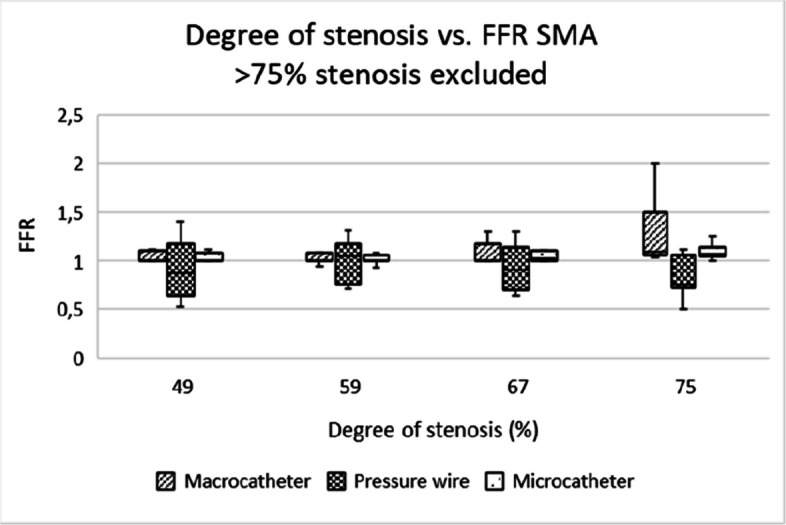
Box-and-whisker plot showing the distribution and variability of FFR measurements across different degrees of stenosis in the superior mesenteric artery, when excluding the 82% and 92% stenosis. FFR = fractional flow reserve, SMA = superior mesenteric artery

**Fig. 7:**
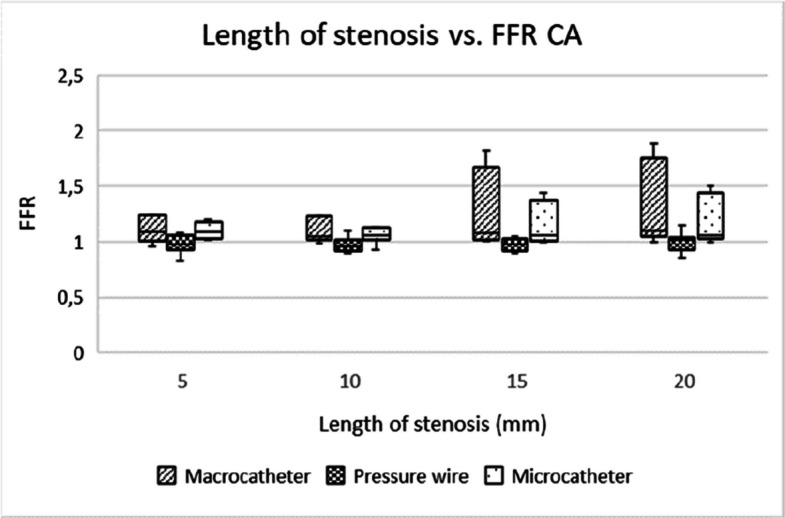
Box-and-whisker plot showing the distribution and variability of FFR measurements across different stenosis lengths in the coeliac artery. FFR = fractional flow reserve, CA = coeliac artery

**Fig. 8:**
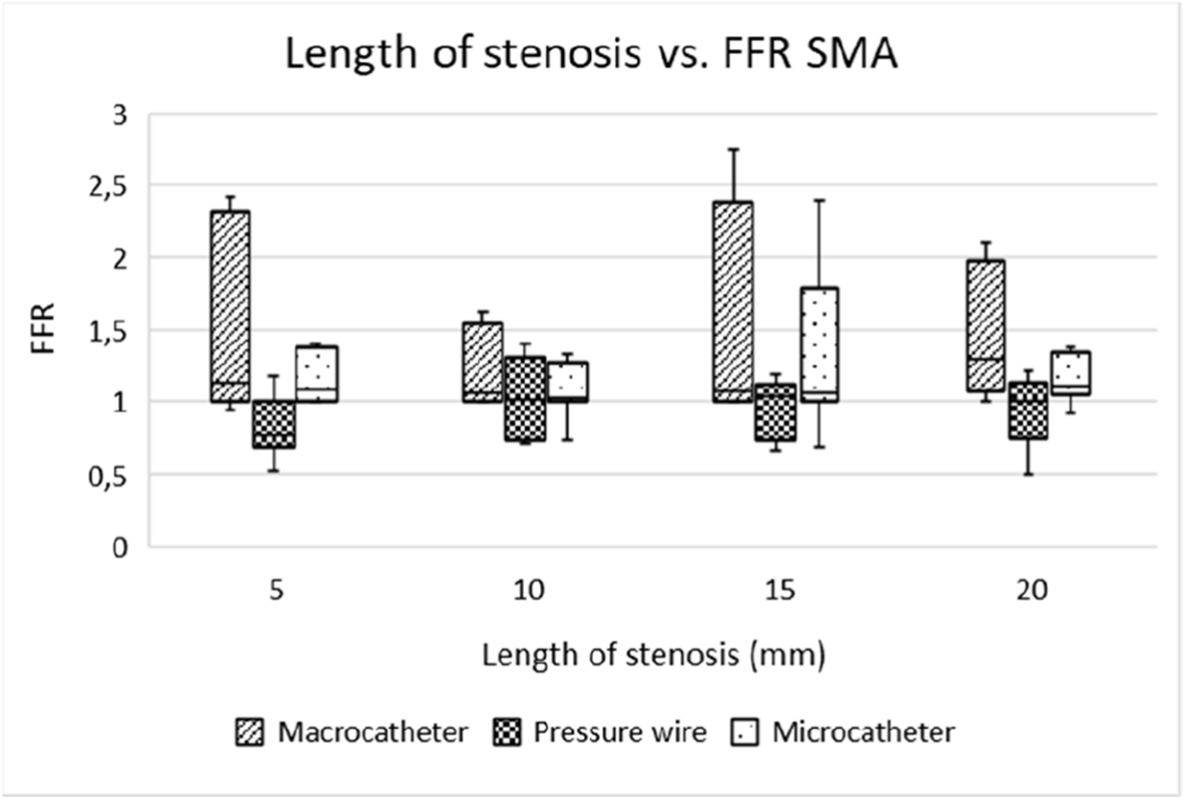
Box-and-whisker plot showing the distribution and variability of FFR measurements across different stenosis lengths in the superior mesenteric artery. FFR = fractional flow reserve, SMA = superior mesenteric artery

**Fig. 9:**
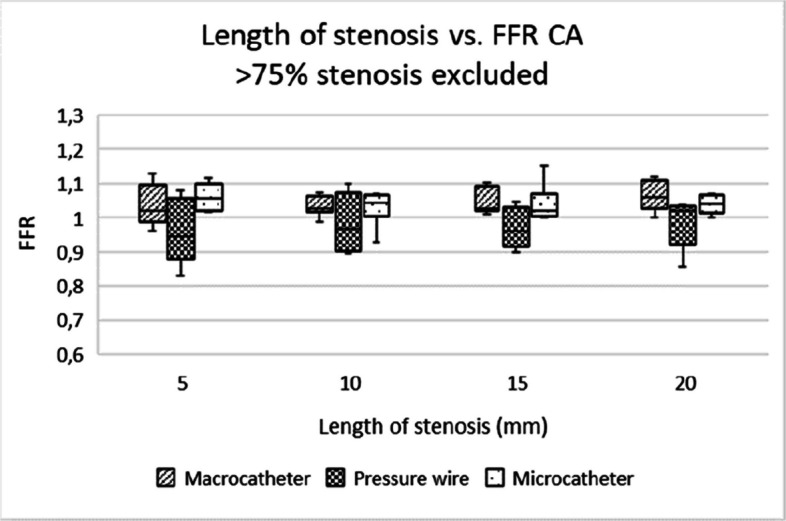
Box-and-whisker plot showing the distribution and variability of FFR measurements across different stenosis lengths in the coeliac artery, when excluding the 82% and 92% stenosis. FFR = fractional flow reserve, CA = coeliac artery

**Fig. 10:**
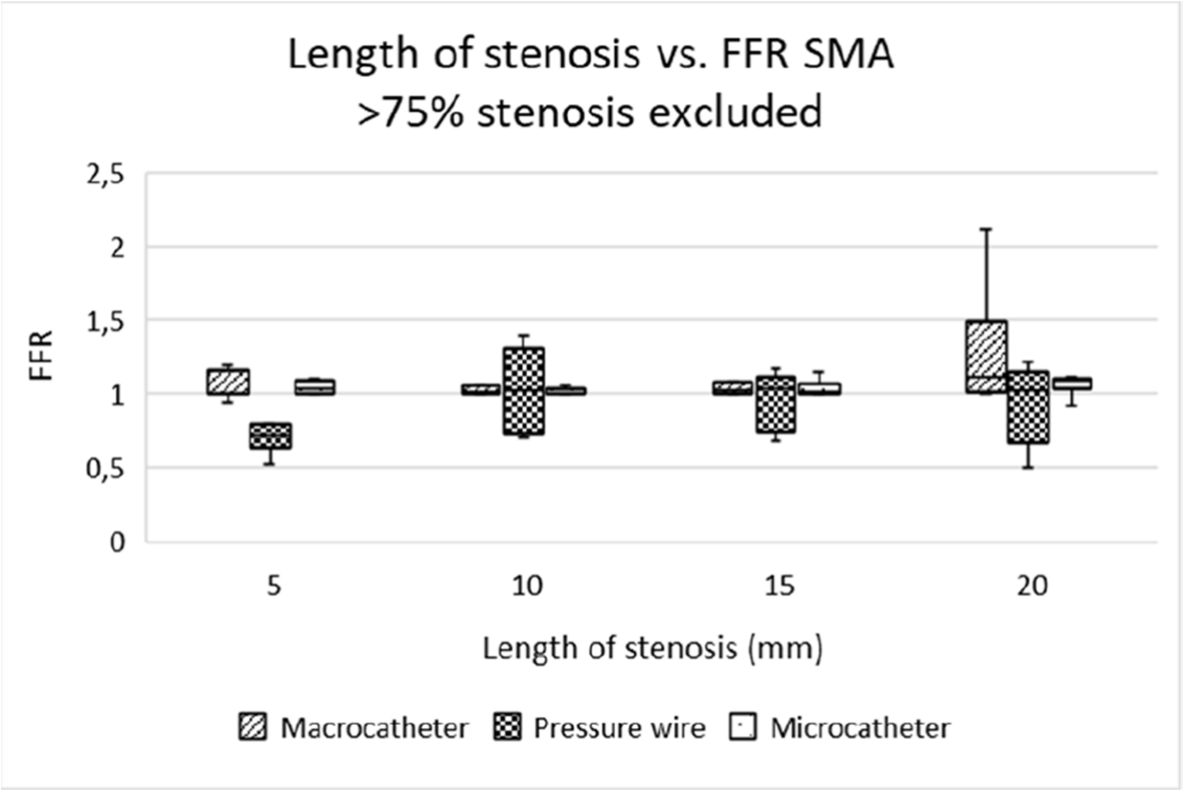
Box-and-whisker plot showing the distribution and variability of FFR measurements across different stenosis lengths in the superior mesenteric artery, when excluding the 82% and 92% stenosis. FFR = fractional flow reserve, SMA = superior mesenteric artery

In vivo studies are needed to validate these findings and explore the clinical implications of pressure measurements in predicting symptom relief following mesenteric artery stenting.

## Data Availability

The datasets are available from the corresponding author on request.
